# Identification of Barriers to Stroke Awareness and Risk Factor Management Unique to Hispanics

**DOI:** 10.3390/ijerph13010023

**Published:** 2015-12-22

**Authors:** Marina Martinez, Nitin Prabhakar, Kendra Drake, Bruce Coull, Jenny Chong, Leslie Ritter, Chelsea Kidwell

**Affiliations:** Department of Neurology, University of Arizona College of Medicine, 1501 N. Campbell Avenue Tucson, AZ 85724, USA; nprabhakar@email.arizona.edu (N.P.); kdrake@neurology.arizona.edu (K.D.); coullb@medadmin.arizona.edu (B.C.); jchong@neurology.arizona.edu (J.C.); lsr@email.arizona.edu (L.R.); ckidwell@neurology.arizona.edu (C.K.)

**Keywords:** stroke, Hispanics, prevention

## Abstract

Barriers to risk factor control may differ by race/ethnicity. The goal of this study was to identify barriers to stroke awareness and risk factor management unique to Hispanics as compared to non-Hispanic whites (NHWs). We performed a prospective study of stroke patients from an academic Stroke Center in Arizona and surveyed members of the general community. Questionnaires included: the Duke Social Support Index (DSSI), the Multidimensional Health Locus of Control (MHLC) Scale, a stroke barriers questionnaire, and a Stroke Awareness Test. Of 145 stroke patients surveyed (72 Hispanic; 73 NHW), Hispanics scored lower on the Stroke Awareness Test compared to NHWs (72.5% *vs.* 79.1%, *p* = 0.029). Hispanic stroke patients also reported greater barriers related to medical knowledge, medication adherence, and healthcare access (*p* < 0.05 for all). Hispanics scored higher on the “powerful others” sub-scale (11.3 *vs.* 10, *p* < 0.05) of the MHLC. Of 177 members of the general public surveyed, Hispanics had lower stroke awareness compared to NHWs and tended to have lower awareness than Hispanic stroke patients. These results suggest that Hispanic stroke patients perceive less control over their health, experience more healthcare barriers, and demonstrate lower rates of stroke literacy. Interventions for stroke prevention and education in Hispanics should address these racial/ethnic differences in stroke awareness and barriers to risk factor control.

## 1. Introduction

Stroke is the leading cause of adult disability and among the top five causes of death in America [1]. Approximately 795,000 strokes occur annually; on average, one stroke occurs every 40 s in the United States [2]. Medically underserved populations, particularly Hispanics (which compose the largest minority group in the nation) are disproportionately affected by stroke with prior population-based studies showing higher incidence rates in Hispanics [3,4,5]. Furthermore, Hispanics are more likely to have a recurrent, or secondary, stroke as compared to their non-Hispanic white (NHW) counterparts [6]. Moreover, minority groups suffer from greater neurological impairment, poorer functional outcomes, and higher mortality rates from stroke [7,8,9,10].

Stroke education and prevention remains the greatest opportunity for decreasing stroke incidence and mortality [11,12]. However, evidence-based therapies for stroke prevention are not properly adopted despite endorsement from current national guidelines [13]. Stroke prevention translates to successful management of cerebrovascular risk factors. This is particularly important in populations, including Hispanics, that have higher incidences of stroke risk factors such as diabetes, hypercholesterolemia, obesity, and physical inactivity, as compared to NHWs [14]. While conflicting data exist regarding the prevalence of hypertension among Hispanics, studies suggest an increase in prevalence over the past decade, regardless of how Hispanic prevalence of hypertension compares to that in whites [14,15,16]. Overall, minority populations are less likely to engage in stroke prevention measures than whites, suggesting the existence of unique barriers specific to minorities in the US [17,18].

Multiple barriers exist that can impede optimal risk factor management in stroke patients. These include barriers in stroke education/literacy, health locus of control, social support, as well as barriers relating to medical knowledge, medications, and healthcare access. Yet, few studies have systematically assessed these barriers to stroke risk factor management in Hispanics. With this study, our aim was to evaluate and identify these potential barriers to risk factor management and stroke prevention in Southern Arizona, with a particular focus on barriers within the Hispanic population. The barriers identified in this proposed study will form the foundation of a community-based and culturally-tailored intervention program that will address stroke risk factor management and prevention of subsequent strokes among Hispanic stroke survivors with the ultimate goal of reducing health disparities in stroke within this population.

## 2. Experimental Section

### 2.1. Subjects and Recruitment

Stroke patients were recruited from Banner-University Medical Center Tucson, an academic stroke center and the main teaching hospital affiliated with the University of Arizona in Tucson, Arizona. Banner-University Medical Center is certified by the Joint Commission as a primary stroke center. All potential patients were identified by the daily log of patients seen by the stroke service. Eligible participants were identified prospectively and approached for participation while in the hospital. Inclusion criteria were as follows: stroke diagnosis abstracted from medical record (ischemic, hemorrhagic, or transient ischemic attack), age ≥18. Only patients that were functionally able to complete the survey were included in this study.

All stroke patients admitted to the hospital receive standardized stroke education during their hospitalization. Patients are given a stroke booklet covering the following topics: ischemic stroke, hemorrhagic stroke, transient ischemic attack; managing risk factors and stroke prevention; improving language skills, coping with difficult swallowing; mood swings and depression; medications; lifestyle changes. In addition to patient education provided by the stroke team physicians, nurses review the booklet with patients on an individual basis, focusing on the risk factors relevant to the patient. Spanish booklets and Spanish-speaking nursing staff are available for patients that do not speak English.

Additionally, members of the general public from Tucson, AZ were recruited for this study and given a modified version of the inpatient survey. General public participants were recruited and completed the surveys at Tucson community events that were expected to attract attendees that were representative of the overall Tucson population. The study protocol was approved by the University of Arizona Institutional Review Board (Project No: 13-0806; Approval date: 12/12/2013) and all aspects of the study were in accordance with the latest revision of the Declaration of Helsinki. All participants provided written informed consent prior to study.

### 2.2. Protocol

The survey included the following components: a demographic questionnaire, the Duke Social Support Index (DSSI), the Multidimensional Health Locus of Control (MHLC) Scale, a questionnaire assessing perceived barriers to healthcare, and a modified Stroke Awareness Test (SAT). The demographic questionnaire included age, gender, and self-reported race (white, black/African American, Asian, Native Hawaiian/Other Pacific Islander, American Indian/Alaska native, or other) and ethnicity (Hispanic *vs.* non-Hispanic). The DSSI quantifies the amount and satisfaction of the stroke patient’s social support and is composed of three separate sections: social score (range: 4–12), satisfaction score (range: 7–21), and total score (range 11–33). The social score quantifies the amount of support, the satisfaction score assesses how content an individual is with the amount of support they have, and the total score is a sum of the two [19]. The MHLC scale assesses the stroke patient’s perceptions of health control [20,21]. Similar to the DSSI, the Multidimensional Health Locus of Control Scale consists of three separate sub-scores each having a score range of 4–16: internal, chance, and powerful others scores. The barriers questionnaire was subdivided to assess three distinct barriers to stroke care: medical knowledge barriers (score range 1–5), medication barriers (score range 3–15), and healthcare access barriers (score range 4–20). The SAT assesses knowledge of stroke symptoms and appropriate course of action in response to signs of a stroke [22]. Percent of items correct were reported for the SAT. Furthermore, a question testing knowledge of tissue plasminogen activator (tPA) was analyzed separately from the SAT. The question is as follows: “Do you know of any medication or treatment that can be given at the time of a stroke to prevent further brain injury?” Answers were considered correct if any of the following answers were given: “tPA”, “a shot/medication/IV that has to be given in time”, or a combination of either of these answers. Surveys for the general public portion of this study included the following: DSSI, Barriers questionnaire, and the SAT.

Surveys were available in English and Spanish. Furthermore, one bilingual research coordinator was available on a full-time basis for Spanish-speaking study participants.

### 2.3. Statistical Analyses

All statistical comparisons were performed using the SPSS statistical software package (v. 23.0; IBM, Armonk, NY, USA). Baseline characteristics and survey results were compared between groups employing Student’s *t*-tests and chi square tests as appropriate. After confirming equality of variance with a Levene’s test, comparisons between Hispanics and non-Hispanic whites were performed using an independent samples Student’s *t*-test with significance set at an α level of 0.05. Student’s *t*-tests were also used for comparisons between the inpatient and general public groups. Chi-square analyses were performed to test group differences for three items (gender, stroke subtype, and the tPA question). Calculation of sample size was performed using G*Power software (v. 3.1; Faul, Erdfelder, Lang, & Buchner; University of Dusseldorf, Dusseldorf, Germany) [23]. This calculation estimated that 140 participants (70 Hispanic and 70 NHW) needed to be recruited for each sub study (stroke and general population) to show a medium effect (d = 0.5, α level *p* = 0.05, power of 80%).

## 3. Results and Discussion

### 3.1. Results 

A total of 145 stroke patients were recruited (72 Hispanic and 73 NHW). Hispanic study participants included a combination of English and Spanish-only speaking individuals. Table 1 shows study participant characteristics by group. Overall, Hispanics were significantly younger than NHWs; however, there was no difference in frequency by gender or stroke type.

**Table 1:** Stroke participant demographics.

Variable	Hispanics (*n* = 72)	NHWs (*n* = 73)	*p*-Value
Age	60.4 ± 13	67.7 ± 13	0.001
Male Gender	31 (43.1%)	36 (49.3%)	0.450
Stroke Type			0.884
Ischemic	48 (69.6%)	42 (67.7%)	
ICH	7 (10.1%)	8 (12.9%)	
TIA	14 (20.3%)	12 (19.4%)	

Table 2 provides the survey results comparing Hispanics *vs* NHWs. Hispanics scored lower on the stroke awareness test compared to NHWs (72.5% correct *vs.* 79.1% correct for Hispanics *vs.* NHWs, respectively, *p* = 0.029). A larger percentage of Hispanic stroke patients was not aware of tPA existing as an acute treatment for stroke compared to NHW inpatients (91.5% *vs.* 79.2%, *p* = 0.036). Hispanics consistently had significantly lower scores for all three barrier categories (medical knowledge barriers, medication barriers, and healthcare access barriers); on this scale lower scores translate to Hispanics experiencing *higher* barriers as compared to their Caucasian counterparts. Hispanics and whites were found to score similarly in the social, satisfaction and total score categories on the Duke Social Support Index. On the Multidimensional Health Locus of Control Scale, Hispanics and NHWs had similar scores for the internal and chance score categories; however, Hispanics had a higher score for the powerful others category (11.3 *vs.* 10 for Hispanics *vs.* NHWs, respectively, *p* = 0.00).

**Table 2:** Stroke participant survey results.

Variable	Hispanics	NHWs	*p*-Value
SAT Percent Correct	72.5%	79.1%	0.029
SAT tPA Knowledge	6 (8.5%)	15 (20.8%)	0.036
Barriers			
Medical Knowledge (Range 1–5)	3.2 ± 1	3.6 ± 1	0.023
Medications (Range 3–15)	9.5 ± 3	11 ± 2	<0.001
Healthcare Access (Range 4–20)	14.8 ± 3	16 ± 2	0.009
DSSI			
Total Score (Range 11–33)	26.61 ± 4	27.71 ± 4	0.089
Social Score (Range 4–12)	8.29 ± 4	8.67 ± 3	0.228
Satisfaction Score (Range 7–21)	17.75 ± 2	18.64 ± 2	0.146
MHLC			
Internal Score (Range 4–16)	11 ± 2	11 ± 2	0.804
Chance Score (Range 4–16)	8.9 ± 2	8.7 ± 2	0.383
Powerful Others Score (Range 4–16)	11.3 ± 2	10 ± 2	<0.001

In the sub-study of 177 members of the general population, a total of 117 Hispanics and 60 NHWs completed the survey. Hispanic and NHWs had similar age and gender representation (Table 3). Hispanic members of the general public scored lower than both NHWs and Hispanic stroke inpatients on the SAT (Table 3 and Table 4). However, there was no difference in stroke awareness between the NHW inpatients and NHW members of the general public (Table 4). General public survey participants scored similarly to stroke inpatients in the Barriers questionnaire regardless of ethnicity (Table 4). Hispanics among the general public have lower stroke awareness and experience higher barriers compared to their NHW counterparts (Table 3).

**Table 3:** Demographics and survey results for general public survey participants.

Variable	Hispanics (*n* = 117)	NHWs (*n* = 60)	*p*-Value
Age	57.7 ± 17	55.7 ± 12	0.451
Male Gender	31 (26.5%)	22 (36.7%)	0.162
SAT Percent Correct	66.6%	76.4%	0.002
Barriers			
Medical Knowledge (Range 1–5)	3.3 ± 1	4.0 ± 1	<0.01
Medications (Range 3–15)	9.3 ± 3	11.7 ± 3	<0.01
Healthcare Access (Range 4–20)	13.9 ± 4	16.5 ± 3	<0.01

**Table 4:** Comparisons between stroke patients and general public participants: Hispanics and NHWs.

Variable	Hispanic-Stroke	Hispanic-General Public	*p*-Value
SAT Percent Correct	75.5%	66.6%	0.056
Barriers			
Medical Knowledge (Range 1–5)	3.2 ± 1	3.3 ± 1	0.603
Medications (Range 3–15)	9.5 ± 3	9.3 ± 3	0.693
Healthcare Access (Range 4–20)	14.8 ± 3	13.9 ± 4	0.174
	NHW-Stroke	NHW-General Public	*p*-Value
SAT Percent Correct	79.1%	76.4%	0.346
Barriers			
Medical Knowledge (Range 1–5)	3.6 ± 1	4.0 ± 1	0.066
Medications (Range 3–15)	10.9 ± 2	11.7 ± 3	0.113
Healthcare Access (Range 4–20)	15.9 ± 2	16.5 ± 4	0.339

### 3.2. Discussion

Overall, the results from this study suggest Hispanic stroke patients experience more barriers to stroke prevention and lower stroke-specific health literacy compared to non-Hispanic whites. An ancillary study of the general public confirmed that these barriers extend to the larger Hispanic population. Some of these barriers may be unique based on cultural and/or ethnic differences.

Health literacy is crucial to management of chronic diseases, including stroke, by influencing healthcare system navigation, treatment-seeking behavior, and adherence to medical treatment [24,25,26,27]. Challenges to health literacy may be more prominent in Hispanics due to their collective education, income, and language profile [14]. In general, minority populations have poor stroke literacy and are less likely to know the signs and symptoms of stroke [28,29,30,31]. In this study, despite equal in-hospital stroke education for both groups, Hispanics were shown to have persistent decreased knowledge of stroke symptoms.

In the sub-study of members of the general public we found that: (1) Hispanics had lower stroke awareness than NHWs, and (2) Hispanic stroke patients had higher knowledge of stroke than Hispanic members of the general Tucson population. This data shows that Hispanics do benefit from inpatient stroke education, albeit not to the same extent as NHWs. Collectively, suggest that further study is necessary to improve stroke patient education and also highlights a potential role for culturally-tailored stroke education materials for all Hispanics (stroke patients and those in the general community setting). Cultural tailoring, defined as “the development of interventions, strategies, messages, and materials to conform with specific cultural characteristics” may make this educational material more effective as it increases the relevance to the receiver and is better at capturing attention and stimulating information processing among targeted audiences. Prior studies have demonstrated the efficacy of culturally tailored health interventions [32,33,34,35,36,37,38,39]. Despite Hispanic stroke inpatients scoring significantly lower than NHWs in the stroke awareness test portion of this survey study, overall both groups could improve on stroke knowledge.

Another important aspect of stroke education is knowledge of acute stroke treatments, particularly administration of tissue plasminogen activator (tPA), a clot dissolving agent that has been shown to improve outcomes when administered within 3–4.5 h of stroke onset. Knowledge of tPA is important as it encourages individuals to call emergency services when stroke symptoms occur. We found that Hispanics had a lower awareness of tPA as an acute stroke treatment than NHWs. However, the knowledge of tPA was surprisingly low for *all* surveyed stroke patients; only 8.5% of Hispanics and 20.8% of NHWs had knowledge of tPA. Again, these results highlight the knowledge deficiencies in both Hispanics and the NHWs, and point to the need for further widespread educational campaigns regarding stroke education.

This study tested for three specific barriers that influence stroke care: general medical knowledge, medication access and cost, and healthcare access in general. In each category, Hispanics reported significantly greater barriers compared to NHWs. These factors are crucial to stroke care as they directly affect vascular risk factor control and stroke prevention measures. Similar to the stroke patients, Hispanic members of the general public perceive more barriers than whites. Though healthcare access is not easily modifiable, the gaps in medical knowledge and medication adherence provide areas for improved education that may benefit Hispanics.

Social support is crucial to risk factor control in any chronic illness, including stroke; for this reason, this study sought to assess the social support structure of stroke patients using the Duke Social Support Index. Positive social support is associated with engagement in medication adherence, self-management behavior, and overall positive health outcomes (including improved blood pressure control), all of which are factors that affect stroke outcomes [40,41,42,43]. In this study, Hispanics and non-Hispanic whites did not differ in social support, either in quantity of, or satisfaction with, their support. Though this is a favorable result, it is an unexpected one considering the long-documented history of familism and allocentrism in the Hispanic population within the United States, which generally translates to elevated social support in this group [44,45]. Though social support is perceived as relatively high in both groups, further research is necessary to explore whether perceived social support might change upon release of the stroke patient to the community setting.

Health locus of control refers to the extent to which an individual perceives their health to be within their own control and is usually classified as either internal or external [46]. An internal locus of control is characterized by the belief that health is within individual control and has been associated with positive health behaviors and outcomes [47,48,49,50,51,52,53]. Our results suggest that Hispanics and NHWs have similar moderate levels of internalization; furthermore, the groups show that they do not believe chance affects their health. Hispanics, however, did score higher on the powerful others score compared to non-Hispanic whites, suggesting that Hispanics rely on others, including friends, family, and their healthcare providers, to a greater extent than NHWs. Despite Hispanics having similar quantity of and satisfaction with their social support as compared to NHWs, it seems that Hispanics rely more heavily on their social circle for health reasons. Therefore, despite the results of the DSSI, incorporation of social support should be a feature in interventions for stroke prevention in Hispanics. Further research is required to clarify the relationship between external locus of control and health behaviors in stroke.

Several limitations exist in our study. Stroke severity was not included as a variable in the analysis; it is possible that results may differ across different stroke severities. Our goal was to include only patients who were able to directly participate in the survey so the population was likely biased towards less severe strokes. Additionally, though information on stroke subtype (TIA, ischemic, or hemorrhagic) was collected, we did not compare results by these subtypes due to small numbers. Furthermore, the research coordinators did not track stroke patients or members of the general public that declined participation or their reasons for declining; therefore, there is a possibility of selection bias in our study.

## 4. Conclusions

In this prospective, single center study of stroke patients in Southern Arizona, stroke survivors who self-identified as Hispanic experience an increased perception of barriers relating to general medical knowledge, medication adherence, and healthcare access, have decreased stroke knowledge (including knowledge of acute stroke therapy), and have higher reliance on “powerful others” for control of their health. Similarly, surveyed Hispanics in the general population of Tucson, AZ display lower awareness of stroke and report perceiving higher barriers compared to NHWs. In the future, these areas can be targeted in culturally-tailored interventions aimed at improving risk factor control and health literacy for stroke prevention in Hispanics, which would ultimately reduce incidence of recurrent stroke in this population.
